# Immune-Related Diarrhea and Colitis in Non-small Cell Lung Cancers: Impact of Multidisciplinary Management in a Real-World Setting

**DOI:** 10.1093/oncolo/oyad238

**Published:** 2023-08-21

**Authors:** Laura Bonanno, Martina Lorenzi, Davide Massa, Mattia De Nuzzo, Valentina Angerilli, Fabiana Zingone, Brigida Barberio, Alberto Russi, Fabio Girardi, Alessandra Ferro, Alessandro Dal Maso, Stefano Frega, Giulia Pasello, Angelo Paolo Dei Tos, Marina Coppola, Matteo Fassan, Edoardo Vincenzo Savarino, Valentina Guarneri

**Affiliations:** Oncology 2, Istituto Oncologico Veneto IOV IRCCS, Padova, Italy; Oncology 2, Istituto Oncologico Veneto IOV IRCCS, Padova, Italy; Department of Surgery, Oncology and Gastroenterology, University of Padova, Padova, Italy; Oncology 2, Istituto Oncologico Veneto IOV IRCCS, Padova, Italy; Department of Surgery, Oncology and Gastroenterology, University of Padova, Padova, Italy; Oncology 2, Istituto Oncologico Veneto IOV IRCCS, Padova, Italy; Department of Surgery, Oncology and Gastroenterology, University of Padova, Padova, Italy; Department of Medicine (DIMED), Surgical Pathology Unit, University of Padova, Padova, Italy; Department of Surgery, Oncology and Gastroenterology, University of Padova, Padova, Italy; Gastroenterology Unit, Azienda Ospedale Università of Padua, Padova, Italy; Department of Surgery, Oncology and Gastroenterology, University of Padova, Padova, Italy; Gastroenterology Unit, Azienda Ospedale Università of Padua, Padova, Italy; Pharmacy Unit, Istituto Oncologico Veneto IOV IRCCS, Padova, Italy; Oncology 2, Istituto Oncologico Veneto IOV IRCCS, Padova, Italy; Oncology 2, Istituto Oncologico Veneto IOV IRCCS, Padova, Italy; Oncology 2, Istituto Oncologico Veneto IOV IRCCS, Padova, Italy; Oncology 2, Istituto Oncologico Veneto IOV IRCCS, Padova, Italy; Oncology 2, Istituto Oncologico Veneto IOV IRCCS, Padova, Italy; Department of Surgery, Oncology and Gastroenterology, University of Padova, Padova, Italy; Department of Medicine (DIMED), Surgical Pathology Unit, University of Padova, Padova, Italy; Pharmacy Unit, Istituto Oncologico Veneto IOV IRCCS, Padova, Italy; Department of Medicine (DIMED), Surgical Pathology Unit, University of Padova, Padova, Italy; Istituto Oncologico Veneto IOV IRCCS, Padova, Italy; Department of Surgery, Oncology and Gastroenterology, University of Padova, Padova, Italy; Gastroenterology Unit, Azienda Ospedale Università of Padua, Padova, Italy; Oncology 2, Istituto Oncologico Veneto IOV IRCCS, Padova, Italy; Department of Surgery, Oncology and Gastroenterology, University of Padova, Padova, Italy

**Keywords:** immune checkpoint inhibitors, immune-related adverse events, immune-mediated diarrhea and colitis, collagenous colitis, immunotherapy rechallenge

## Abstract

**Introduction:**

Immune-related adverse events (irAEs) constitute a challenge in the clinical management of solid tumors. This study aims to collect real-world data on the occurrence of immune-mediated diarrhea and colitis (IMDC) in advanced non-small cell lung cancer (aNSCLC) treated with immune checkpoint inhibitors (ICIs) and to assess the clinical impact of a multidisciplinary approach (MDA) on IMDC management.

**Methods:**

We retrospectively collected data on patients with aNSCLC consecutively treated with ICIs, either as single agent or in combination with chemotherapy, between September 2013 and July 2022. Among patients developing IMDC, we conducted blinded revision of colonic biopsies and evaluated the clinical impact of the introduction of MDA through predefined indicators.

**Results:**

Among the 607 patients included, 84 (13.8%) experienced IMDC. Pathological review highlighted a high prevalence of microscopic colitis (28%), with a collagenous pattern linked to longer symptoms duration (*P* = .01). IMDC occurred more frequently in females (*P* = .05) and PD-L1 expressors (*P* = .014) and was correlated with longer progression-free survival (17.0 vs 5.8, *P* < .001) and overall survival (28.3 vs 9.5, *P* < .001). The introduction of MDA was associated with increased employment of diagnostical tools such as fecal calprotectin test (*P* < .001), colonoscopy (*P* < .001), and gastroenterological evaluation (*P* = .017) and a significant decrease in both grade 3 conversion rate (*P* = .046) and recurrence after rechallenge (*P* = .016). Hospitalization rate dropped from 17.2% to 3.8% (*P*: ns).

**Conclusion:**

These findings highlight the clinical relevance of IMDC and support the incorporation of a MDA to optimize the clinical management of this irAE to improve patient care. Prospective validation has been planned.

## Teaser

Immune-related adverse events are a challenge in the clinical management of solid tumors. This study collected real-world data on the occurrence of immune-mediated diarrhea and colitis in patients with advanced non-small cell lung cancer treated with immune checkpoint inhibitors and assessed the clinical impact of a multidisciplinary approach on the management of this adverse event.

**Implications for Practice**

The management of immune-mediated diarrhea and colitis (IMDC) in patients treated with immune-checkpoint inhibitors can constitute a clinical challenge. Our study highlights the clinical relevance of IMDC in real-world patients with non-small cell lung cancer and depicts the clinical impact of introduction of multidisciplinary approach in their management by using predefined indicators. The results support the incorporation of a multidisciplinary approach to optimize clinical management of IMDC and ultimately improve patient care.

## Introduction

The introduction of anti-programmed death (PD)-1/PD-ligand (PD-L) 1 immune-checkpoint inhibitors (ICIs) in clinical management of advanced non-small cell lung cancer (aNSCLC) has significantly improved patients’ overall survival (OS) and quality of life.^1-5^ ICIs’ administration can lead to the development of immune-related adverse events (irAEs) as a consequence of non-specific activation of the immune system and immune-mediated damage, potentially affecting every organ and apparatus, more frequently skin, endocrine system, gastrointestinal tract, and liver.^6^ Although the global incidence of specific irAEs was relatively low in all pivotal studies^1-5,7,8^ and severity presentation was mainly mild to moderate, the management requires prompt recognition and proper treatment in order to avoid prolonged, severe, and even fatal outcomes, which are also associated with high hospitalization rates.^9-13^

Moreover, patients treated in randomized clinical trials (RCTs) are not representative of real-world population due to restrictive selection criteria applied regarding age, performance status (PS), presence of CNS metastasis, comorbidities,^14^ while the use of immunotherapy in “frail” population is increasing.^15^ In parallel, the introduction of immunotherapy in locally advanced and early-stage disease further underlines the importance of proper management of irAEs, while specifically addressed studies are still awaited.

Diarrhea is one of the most common and severe irAEs with an any grade incidence of 8-14% for anti-PD-1 drugs (nivolumab, pembrolizumab, and atezolizumab) and approximately 30% for pembrolizumab combined with chemotherapy. Grades 3-4 diarrhea are reported between 1% and 4% for anti-PD1 monotherapy and 5.2% for combination therapy.^1-5,16^ Diarrhea is the most common symptom of colitis, which can be associated with abdominal pain, distension, hematochezia and mucus in stools. Diarrhea and colitis co-occurrence are collectively referred to as immune-mediated diarrhea and colitis (IMDC).^17^ Median time to symptoms onset is approximately 1 month for anti-CTLA4 drugs and longer for PD-1/PD-L1 agents.^18,19^ According to international guidelines, IMDC management is mainly based on ICI temporary discontinuation, supportive care and corticosteroids therapy; multidisciplinary involvement is encouraged, while clear indication on ICIs reassumption in case of moderate-severe IMDC and management of steroids’ refractory cases are missing.^17,20-23^ In parallel, biomarkers for predicting the risk of toxicity onset, its severity and risk of relapse are not available in clinical setting.^24^

The aim of this study is to describe the incidence, clinical and pathological presentation, management, and outcome of IMDC in a real-world (RW) setting, by describing a large mono-institutional experience including patients with aNSCLC treated with immunotherapy or chemo-immunotherapy. Moreover, we evaluated the impact of the systematic introduction of multidisciplinary evaluation on IMDC management and outcome. The impact of management change was evaluated according to predefined clinical indicators and budget impact analysis.

## Material and Methods

### Patients

The study included a retrospective cohort of patients with cytological or histological diagnosis of advanced NSCLC (stage III, not suitable for radical treatment, and IV according to eighth edition of the TNM Classification of Malignant Tumors) and consecutively treated with anti-PD1 or anti-PD-L1 in any therapeutic line (in monotherapy or combination with chemotherapy) at Veneto Institute of Oncology (Istituto Oncologico Veneto-IOV), from September 2013 to July 2022. Since June 2017, patients with PDL-1 ≥ 50% received pembrolizumab in first line. Since January 2020, patients with PDL-1 0%-49% received the combination of pembrolizumab plus platinum-pemetrexed, according to national guidelines. All patients included had a minimum follow-up of 3 months.

All patients were treated according to clinical practice and since May 2021 thoracic oncologist at IOV started to discuss IMDC cases with gastroenterologists and pathologists specialized in inflammatory bowel diseases and other immune-mediated conditions of the gastrointestinal tract.

Clinical features and radiological imaging of all patients included were reviewed. Details about treatment administration, clinical outcome, incidence, severity, and management of irAEs were collected. In patients who presented diarrhea, signs and symptoms, diarrhea grade at onset and its maximum grade experienced, time to adverse events occurrence and resolution, treatment discontinuation and resumption of immunotherapy were collected. Moreover, serologic, fecal, or diagnostic evaluations such as colonoscopy were registered. In addition, colonic biopsies with related pathological characteristics were collected and blindly reviewed by pathologists with expertise in gastrointestinal disease evaluation. When required, the type, starting dose, maximum dose and duration of corticosteroids or other immunosuppressive therapies, were evaluated. The adjudication of the symptoms as immune-mediated were reviewed from the clinicians of the study team, taking into account the clinical diagnosis indicated by the treating-physicians, clinical features reported and the results of the diagnostic work-up and treatment used. Per internal procedure, all patients treated at our institution and included in the study were instructed to report symptoms onset as soon as possible and had a direct way to inform the clinicians (phone number, email).

This study was conducted in accordance with Good Clinical Practice guidelines and the Helsinki declaration. All the participants signed the specific Informed Consent Form, according to the Regulation (EU) 2016/679 of the European Parliament and the Council on personal data protection. The study was approved by the IOV Ethical Committee (292, 13 May 2019).

### Pathological Evaluation

All available endoscopic biopsies of the colon-rectum were reviewed by 2 gastrointestinal pathologists (V.A. and M.F.). Biopsies were fixed in 10% buffered formalin, embedded in paraffin, sectioned at 5 mm thickness, and stained with hematoxylin and eosin. Where available, immunohistochemical stains were reviewed. The presence or absence of the following histopathologic features was assessed: crypt atrophy/loss, crypt distortion, mucin depletion, apoptotic bodies, lamina propria expansion, crypt abscess, subepithelial macrophages, superficial erosion/ulceration, ischemic colitis-like features, Paneth metaplasia. The following histopathologic features were assessed semiquantitatively: intraepithelial lymphocytes count (absent; 0-2/100 enterocytes; 3-20/100 enterocytes or > 20/100 enterocytes), lymphomonocitic infiltrate (absent, mild, moderate or heavy), granulocytic infiltrate (absent, mild, moderate or heavy), cryptis (absent, focally present). A global score was also given based on the number and severity of the colitis-associated histopathologic features.

### Statistical Analyses

The primary objectives of the study were to describe the incidence and management of IMDC in a real-world scenario and to evaluate the impact of multidisciplinary discussion on their diagnostic-therapeutic management.

Some key indicators were identified to synthetize IMDC management and outcome and observe changes before and after the introduction of multidisciplinary discussion. Indicators of outcome were maximum grade of IMDC reported, the rate of conversion from grades 1-2 to grade ≥3 IMDC, the rate of hospitalization for IMDC and the rate of relapse of symptoms after re-assumption of immunotherapy. We used G3 conversion to refer to all cases in which symptoms worsened during active management (after reported onset), thus excluding all cases reported as G3 at baseline. Indicators of diagnostic-therapeutic pathway considered were: the proportion of patients who underwent endoscopy and biopsy, the use of fecal calprotectin test in clinical practice, the ratio of definitive interruption for irAEs, the frequency of ICIs rechallenge after irAE resolution.

Median progression-free survival (mPFS) was calculated from the date of initiation of study treatment until radiological progression of disease or death from any cause; median OS (mOS) was calculated as the time from the date of initiation of treatment to the date of death from any reason. Radiological response was evaluated according to the RECIST criteria v.1.1 (Response Evaluation Criteria in Solid Tumors). The response rate (RR) consists of the proportion of patients obtaining partial response or complete response following immunotherapy. The disease control rate (DCR) refers to the number of patients obtaining partial response, complete response, and disease stability. The severity of the ir.AEs was defined according to the Common Terminology Criteria for Adverse Events (CTCAE), version 5.0. The Kaplan-Meier method was used to estimate mPFS and mOS, while the chi-square and the Fisher exact test were used to compare proportions. The chi-square, Mann-Whitney or Fisher exact test, and multiple logistic regression were used for correlation analysis. Statistical analysis was performed through Sigma-Plot (version 11; Systat Software, San Jose, CA).

In order to assess the budget impact of the organizational changes in the management of IMDC, a total cost analysis was conducted. Over the period during which one of these toxicities was detected (from the date of onset to the date of resolution of symptoms), we reported the following costs: i) cost of purchasing budesonide and/or immunomodulatory drugs (infliximab/vedolizumab), ii) cost of performing fecal calprotectin test, iii) cost of performing colonoscopy, and iv) overall cost of hospitalization. The latter costs were calculated using the tariff system of inpatient hospitalization services used in Italy (DRG).

The analysis was conducted by comparing the total costs between before and after the start of the change in multidisciplinary management of IMDC. The period being analyzed was between January 2017 and May 2022 and the cut-off date used was May 2021.

## Results

### Study Population and Outcomes

At data cut-off (August 2022), a total of 607 advanced patients with NSCLC treated with an anti-PD1/PD-L1 agent were included. Patients’ characteristics are summarized in Supplementary Table S1.

The median age was 68.7 years, most patients were male (*n* = 389, 65.5%), current (*n* = 192, 31.6%), or former smoker (*n* = 307, 50.5%). Adenocarcinoma histology was detected in 394 cases (64.9%); PD-L1 (tumor proportion score—TPS) status was assessed in most patients (*n* = 518, 85.3%) and its expression was over 50% in 219 cases (36.1%). The majority of patients had stage IV disease (*n* = 599, 98.7%) and lower than 3 metastatic sites at diagnosis (*n* = 467, 76.9%). Five hundred and eight (83.7%) patients were assigned a score of 0 or 1 using Eastern Cooperative Oncology Group Performance status (ECOG-PS).

ICIs were administered as single-agents in 483 (79.6%) patients and pembrolizumab was the most frequent one (*n* = 233, 38.4%); 50.7% of patients received ICIs as first-line treatment, both in monotherapy (*n* = 184, 30.3%) and in combination with chemotherapy (*n* = 124, 20.4%).

Patients’ outcome endpoints according to the treatment received are detailed in Supplementary Table S2. ICIs administration was associated with an RR of 30% (95% CI 26.4-33.8) and a DCR of 59.5% (95% CI, 55.5-63.4%). RR was statistically higher when ICIs were administered in first-line setting, both as monotherapy (*n* = 79, 49.2%, 95% CI, 35.7-50.4%, *P* < .001) and in combination with Chemotherapy (*n* = 96, 50.8%, 95% CI, 41.7-59.9, *P* < .001). After a median follow-up of 8.8 (inter-quartile ratio, IQR, 3.5-17.7) months, mPFS in the overall population was 7.7 (95%CI, 6.6-8.9) months and mOS was 10.9 (95% CI: 9.4-12.4)). Patients receiving ICIs in first-line setting had significantly higher PFS (*P* < .001) and OS (*P* < .001; Supplementary Table S2).

Clinico-pathological features affecting outcome of patients treated with ICIs both in terms of OS and PFS were PD-L1 expression, the number of metastatic sites at diagnosis and PS (Supplementary Table S3). Adenocarcinoma histology was associated with improved OS but not affected PFS following immunotherapy (Supplementary Table S3).

### Incidence and clinical-pathological features associated with IMDC in a real-world setting

In our cohort, 111 patients (18.3%) experienced diarrhea of any grade. In 84 cases (75.7%), diarrhea was judged as immune related, on the basis of clinical presentation (onset timing, duration, resolution of symptoms) and exclusion of infectious causes by using standard diagnostic work-out. All cases of diarrhea considered as non-immune related had their simptoms resolved within 3 days from onset, without immune-suppressive treatments or delay of ICIs administration. IMDC occurred in 19 out of 124 (15.3%) patients receiving ICIs plus chemotherapy and in 65 out of 483 (13.5%) patients receiving ICIs monotherapy (*P* = .696; Supplementary Table S4).

Median time to diarrhea onset was 3.3 (IQR 1.530-7.32) months. In 77 (91.7%) cases, diarrhea was of grades 1-2 at onset, but in 14 (16.7%) patients, symptoms worsened afterward, and irAE was later reported as grades 3-4. Associated symptoms such as abdominal pain (*n* = 26, 31.0%), bloody diarrhea (*n* = 6, 9.4%), fever (*n* = 2, 2.4%), nausea/vomiting (*n* = 8, 12.5%), weight loss (*n* = 7, 8.3%), and epigastric pain (*n* = 5, 7.3%) were reported by 34 (40.5%) patients. Supplementary Table S4 reports clinical features of patients who experienced IMDC according to the type of treatment received (chemotherapy *plus* ICIs *versus* ICIs monotherapy). No statistically significant differences were identified when comparing the 2 treatment groups.

In our cohort, IMDC was more frequent in female (*P* = .002), in patients with positive (≥1%) PD-L1 expression (*P* = .019), good PS (*P* = .002), and adenocarcinoma histology (*P* = .049). Multivariate analysis confirmed gender (OR 0.478, 95% CI 0.286-0.798, *P* = .005) and PD-L1 status (OR 0.475, 95% CI 0.262-0.861, *P* = .014) as independent risk factors for IMDC occurrence (Table 1).

**Table 1.** Clinico-pathological features associated with immune-mediated diarrhea and colitis (IMDC)

Variable	All population *N* (%)	IMDC *N* (%)	No IMDC *N* (%)	Univariate analysis		Multivariate analysis	
				*P*	OR (95% CI)	*P*	OR (95% CI)
Number of cases	607 (100)	84 (100)	523 (100)				
Age							
<68 years (median)	283 (46.6)	39 (46.4)	244 (46.7)	.937	0.991 (0.624-1.573)		
>68 years (median)	324 (53.4)	45 (53.6)	279 (53.3)				
*Gender*							
Male	398 (65.6)	42 (50)	356 (68.1)	**.002**	**0.469 (0.295-0.747)**	**.005**	**0.478 (0.286-0.798)**
Female	209 (34.4)	42 (50)	167 (31.9)				
*Smoking status*							
Never smokers	92 (15.1)	17 (20.2)	75 (14.3)	.217	1.516 (0.844-2.723)		
Former/current smokers	515	67 (79.8)	448 (85.7)				
*Histology*							
Adenocarcinoma	394 (64.9)	63 (75.0)	331 (63.3)	**.049**	**1.740 (1.030-2.941)**	.108	1.621 (0.899-2.924)
Squamous and other carcinoma	213 (35.1)	21 (25.0)	192 (36.7)				
*Number of metastatic sites*							
0-1	264 (43.5)	43 (51.2)	221 (42.3)	.157	1.433 (0.903-2.274)		
>1	343 (56.5)	41 (48.8)	302 (57.7)				
*Lombo-sacral RT*							
Yes	70 (11.5)	11 (13.1)	59 (11.3)	.735	1.203 (0.602-2.403)		
No	537 (88.5)	73 (86.9)	464 (88.7)				
*PS ECOG at treatment start*							
0-1	510 (84.0)	77 (91.7)	433 (82.8)	**.002**	**2.132 (1.338-3.396)**	.087	2.065 (0.901-4.730)
>1	97 (16.0)	7 (8.3)	90 (17.2)				
PD-L1							
<1%	180 (29.7)	16 (19.0)	164 (31.4)	**.019**	**0.483 (0.268-0.868)**	**.014**	**0.475 (0.262-0.861)**
≥1%	339 (55.8)	57 (67.9)	282 (53.9)				
Not valuable	88 (14.5)	11 (13.1)	77 (14.7)				

Abbreviations: *N*, number of cases; PD-L1, programmed death-ligand 1; PS, performance status; ECOG, Eastern Cooperative Oncology Group; ICIs, immune-checkpoint inhibitors; RT, radiotherapy.

**Figure 1.**
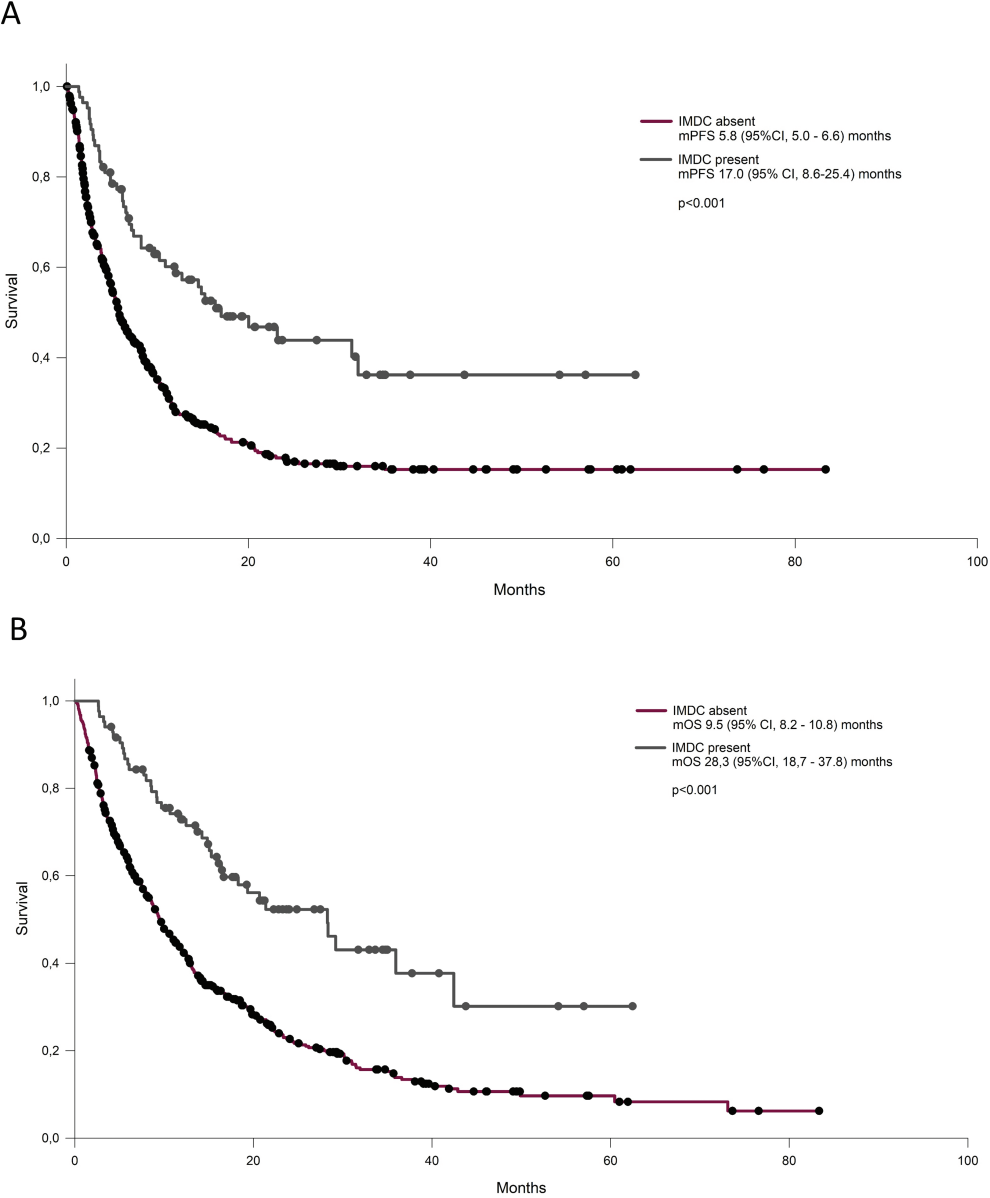
Outcome of patients treated with ICIs according to the presence of immune-related diarrhea in terms of progression-free survival (PFS, A) and overall survival (OS, B).

We also evaluated the impact of IMDC occurrence on patients’ outcome. Patients experiencing immune-related colitis showed a significantly longer PFS (mPFS 17.0, 95% CI 8.6-25.4, versus 5.8, 95% CI 5.0-6.6 months, *P* < .001) and OS (mOS 28.3, 95% CI 18.7-37.8 versus 9.5, 95% CI 8.2-10.8 months, *P* < .001) compared with patients not experiencing IMDC, as depicted in Fig. 1. Multivariate analysis confirmed PD-L1 status, histology, number of metastatic sites at diagnosis, PS and IMDC as factors independently associated with PFS and OS (Supplementary Table S3). To evaluate the impact of a potential immortal time bias, we conducted landmark survival analysis including only patients alive or not progressed at 12-weeks from first ICI administration, which provided consistent results regarding mPFS (*n* = 412, 23.2 vs 10.1 months in IMDC vs no-IMDC, *P* < .001; HR 0.54 [95% CI 0.38-0.77], *P* < .001) and mOS (*n* = 489, 28.4 vs 12.9 months in IMDC vs no-IMDC, *P* = .001, HR 0.50 [95% CI 0.35-0.70], *P* = .001: Supplementary Fig. S1).

### IMDC Management

Diarrhea was the cause of treatment interruption in 59 (70.2%) patients. Sixty-eight patients out of 84 (81.0%) received steroid therapy, 10 of them (11.9%) intravenously; all patients not treated with steroids experienced grade 1 diarrhea. A quarter of patients (*n* = 22, 25.0%) needed other intravenous support. Median steroid-duration time was 122.5 days (IQR 75.0-252.0). One patient (1.2%) received biologic therapy (vedolizumab) in order to control symptoms, after 182 days of steroids treatments. Diagnostic colonoscopy was performed in 30 (30.7%) cases with a median time since symptoms report of 1.9 months (IQR 0.9-4.2).

Importantly, 11 (13.1%) patients required hospitalization for diarrhea-related causes, with a median time for discharge of 15.5 (IQR 9.0-17.0) days. The cause of hospitalization was symptoms severity in 7 cases (63.6%), diverticulitis in 1 case (9.0%), dehydration (*n* = 1, 9.0%), and bone fracture following presyncopal symptoms (*n* = 1, 0.9%). No toxic deaths were registered.

Symptoms resolution was achieved in 81 out of 84 cases (96.4%) at the data cut-off. The median time to symptoms resolution was 48 (IQR 38.7-59.3) days. A trend toward a longer duration of symptoms was reported in patients treated with ICIs *plus* chemotherapy versus ICI alone (median 59 versus 45 days, *P* = .174). After symptoms resolution, treatment was resumed in 30 cases (35.7%); of these, 7 cases (23.3%) had a grade 3 diarrhea. Only 2 patients (6.0%) resumed treatment after disease progression. Median time to treatment resumption was 4.9 months (95%CI −0.4, 10.2). Recurrence of diarrhea was registered in 19 out or 30 (63.3%) cases who resumed treatment with a median time of onset since treatment resumption of 2.1 (95% CI, 1.1-3.0) months.

In our cohort of patients, treatment interruption did not affect outcome either in terms of PFS or OS (Supplementary Fig. S2).

When we compared clinical features and management according to anti-cancer treatment received (mono-immunotherapy versus combination treatment), we noticed no statistically significant difference (Supplementary Table S4).

### Impact of Introduction of a Multidisciplinary Team in Diagnostic-Therapeutic Pathways and Outcomes

Since May 2021, IMDC cases were discussed via email and webinar with gastroenterologists specialized in inflammatory bowel disease and other immune-mediated disorders of the gastrointestinal tract. Moreover, cases undergoing biopsy were discussed with 2 gastrointestinal specialized pathologists. Only selected cases underwent a gastroenterological visit.

**Figure 2.**
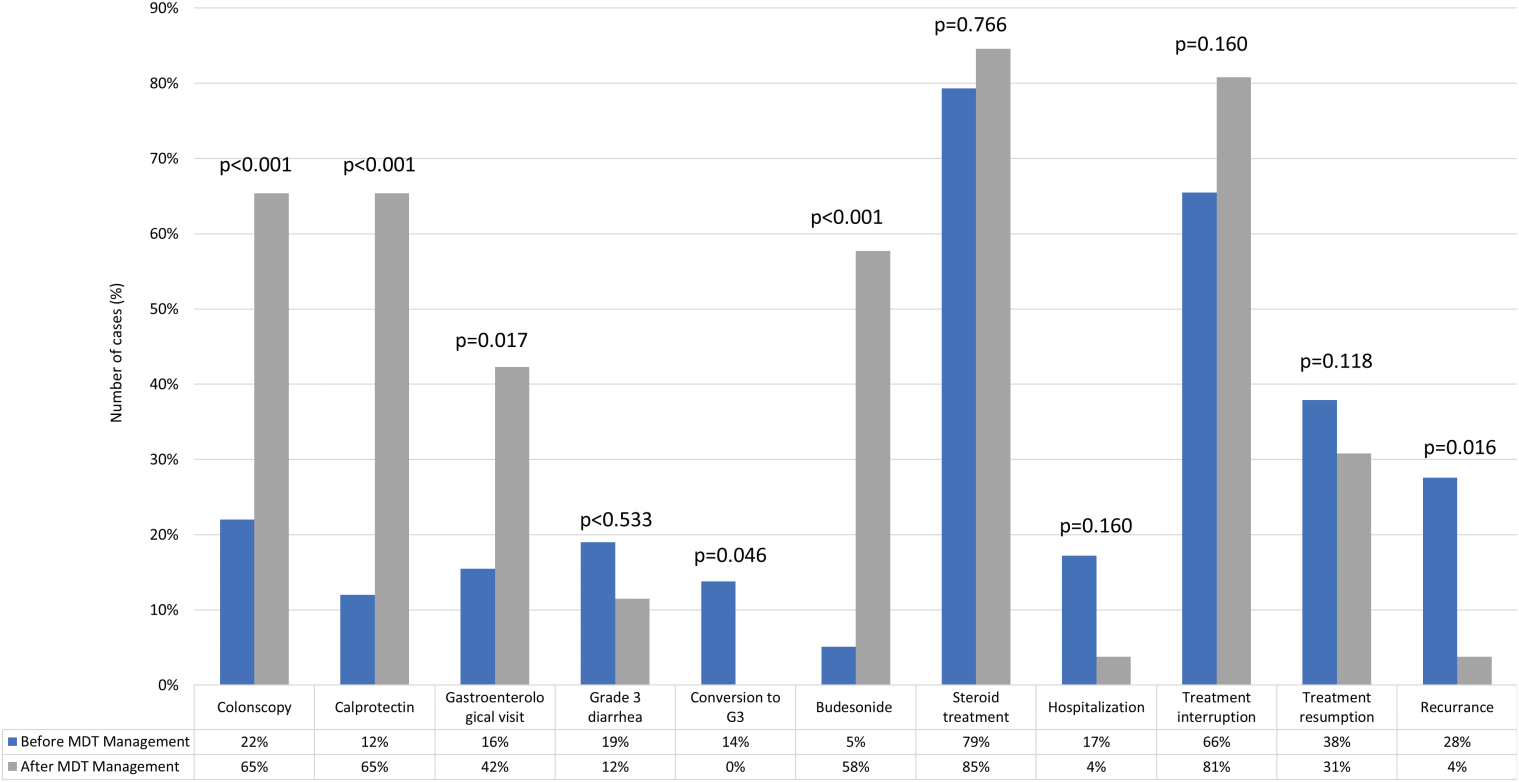
The figure summarized the changes in management and outcome of immune-related diarrhea and colitis (IMDC) after the introduction of multidisciplinary discussion. *In conversion to G3 group were included cases experiencing worsening of symptoms during active management.

We evaluated the impact of multidisciplinary discussion on IMDC management by using predefined pathway indicators (Table 2, Fig. 2).

**Table 2.** Management of immune-mediated diarrhea and colitis (IMDC) before and after the introduction of multidisciplinary team.

Variable	Before MDT collaboration (before May 2021) *N* (%)	After MDT collaboration (after May 2021) *N* (%)	*P*
All cases	58 (100)	26 (100)	
*Treatment*			
ICI plus ChT first Line	10 (17.2)	9 (34.6)	.105
ICI monotherapy	48 (82.8)	17 (65.4)	
*Fecal calprotectin test*			
Yes	7 (12.0)	17 (65.4)	**<.001**
No	51 (87.9)	9 (34.6)	
*Colonoscopy*			
Yes	13 (22.4)	17 (65.4)	**<.001**
No	45 (75.9)	9 (34.6)	
*Gastroenterological visit*			
Yes	9 (15.5)	11 (42.3)	**.017**
No	49 (84.5)	15 (57.7)	
*Grade at onset*			
G1-G2	54 (93.1)	23 (88.5)	.671
G3	4 (6.9)	3 (11.5)	
*Max grade*			
G1	30 (51.7)	10 (38.4)	.374
G2	17 (19.3)	13 (50.1)	
G3	11 (19.0)	3 (11.5)	
Conversion from G1-2 to G3*			
No	50 (86.2)	26 (100.0)	**.046**
Yes	8 (13.8)	0 (0.0)	
*Symptoms duration (days)*			
Median (IQ range)	51.0 (24.0-91.0)	47.0 (24.0-89.8)	.710
*Time to colonoscopy (days)*			
Median (IQ range)	82.0 (34.5-160.0)	55.0 (26.8-120.0)	.438
*Budesonide treatment*			
Yes	3 (5.1)	15 (57.7)	**<.001**
No	55 (94.8)	11 (42.3)	
*Steroid treatment*			
Yes	46 (79.3)	22 (84.6)	.766
No	12 (20.7)	4 (15.4)	
*Steroid duration (days)*			
Median (IQ range)	111.5 (58.0-247.0)	139.5 (84.5-286.5)	.499
*Systemic steroid duration (days)*			
Median (IQ range)	98.5 (43.0-170.0)	121.0 (81.0-198.0)	.342
*Hospitalization*			
Yes	10 (17.2)	1 (3.8)	.160
No	48 (82.8)	25 (96.2)	
*Treatment interruption*			
Yes	38 (65.5)	21 (80.8)	.248
No	20 (34.5)	5 (19.2)	
*Treatment resumption*			
Yes	22 (37.9)	8 (30.8)	.118
No	16 (27.6)	13 (50.0)	
Not applicable	20 (34.5)	5 (19.2)	
*Recurrence*			
Yes	16 (27.6)	1 (3.8)	**.016**
No	6 (10.3)	7 (26.9)	
Not applicable	36 (62.0)	18 (69.2)	
*Biological drug*			
Yes	0 (0.0)	1 (3.8)	.310
No	58 (100)	25 (96.2)	

Abbreviations: MDT, multidisciplinary team; *N*, number; ICI, immune checkpoints inhibitors; ChT, chemotherapy, G, grade; IQ, inter-quartile. *In conversion to G3 group were included cases experiencing worsening of symptoms during active management.

As depicted in Fig. 2, after the introduction of a specialized multidisciplinary team, we report a statistically significant increase in the employment of diagnostical tools such as calprotectin fecal test (12.0% vs 65.4%, *P* < .001), colonoscopy (22.4% vs 65.4%, *P* < .001), and gastroenterological evaluation (15.5% vs 42.3%, *P* = .017).

No statistically significant difference in maximum grade was reported before and after the introduction of the team, anyway after the MTD introduction, no cases worsened during active management and all grade 3 cases were already present at onset (*P* = .046). Interestingly, also the reduction in the toxicity recurrence after treatment resumption (3.8% vs 27.6%, *P* = .016) was statistically significant (Fig. 2, Table 2).

The improvement in diagnostic-therapeutic pathways are likely to lead to customization of management. In particular, re-introduction of ICIs was associated with calprotectin fecal test monitoring and in 20% of patients reintroducing ICIs, only non-systemic steroids were maintained at the time of ICI reintroduction.

As we observed a numerically lower rate of hospitalization after the introduction of multidisciplinary team discussion (17.2 vs 3.8%, *P* = .16), we have conducted a logistic regression analysis to assess the impact of multidisciplinary approach on the risk of hospitalization. We report no statistically significant association after adjusting for gender, PD-L1 status (cut-off 1%), type of treatment (ICI or combination treatment), PS and maximum grade of toxicity (odds ratio OR 0.82: 95% CI 0.14-4.94), However, the direction of the OR may suggest a trend toward a lower risk of hospitalization after the introduction of the multidisciplinary discussion (data not shown). Further details are summarized in Table 2.

### Endoscopic and Pathological Evaluation

Thirty patients (30.7%) underwent colonoscopy, following grade 3 IMDC in 6 cases (20%), grade 2 in 13 (43.3%), and grade 1 symptoms in 11 (36.7%). In 11 (36.7%) patients, macroscopic alterations were observed at endoscopy; in 19 cases (63.3%) random multiple biopsies were performed even in the absence of macroscopic alterations.

Table 3 reports the pathological features assessed in the 25 patients (83.3%) who underwent endoscopic evaluation and had histological material available for revision.

**Table 3.** Microscopic features of available biopsies of patients experiencing immune-mediated diarrhea and colitis.

Variable	*N* (%)
Number of cases	25 (100.0)
*Crypt atrophy/loss*	
Present	12 (48.0)
Absent	13 (52.0)
*Crypt distortion*	
Present	15 (60.0)
Absent	10 (40.0)
*Mucin depletion*	
Present	20 (80.0)
Absent	5 (20.0)
*Apoptotic bodies*	
Present	19 (76.0)
Absent	6 (24.0)
*Lamina propria expansion*	
Present	21 (84.0)
Absent	4 (16.0)
*Collagenous band*	
Absent	16 (64.0)
Focal	5 (20.0)
Extensive	4 (16.0)
*Intraepithelial lymphocytes*	
Absent	1 (4.0)
0-2/100 enterocytes	11 (44.0)
3-20/100 enterocytes	13 (52.0)
>20/100 enterocytes	0 (0.0)
*Lymphomonocytic infiltrate*	
Absent	0 (0.0)
Mild	8 (32.0)
Moderate	17 (68.0)
Heavy	0 (0.0)
*Granulocyte infiltrate*	
Absent	13 (52.0)
Mild	11 (44.0)
Moderate	1 (4.0)
Heavy	0 (0.0)
*Cryptitis*	
Absent	20 (80.0)
Focally present	3 (12.0)
Focally present	2 (8.0)
*Crypt abscess*	
Present	2 (8.0)
Absent	23 (82.0)
*Subepithelial macrophages*	
Present	6 (24.0)
Absent	19 (76.0)
*Superficial erosion/ulceration*	
Present	5 (20.0)
Absent	20 (80.0)
*Ischemic colitis like*	
Present	6 (24.0)
Absent	19 (76.0)
*Paneth metaplasia*	
Present	7 (28.0)
Absent	18 (72.0)
*Global score*	
1	9 (36.0)
2	9 (36.0)
3	7 (28.0)

**Figure 3.**
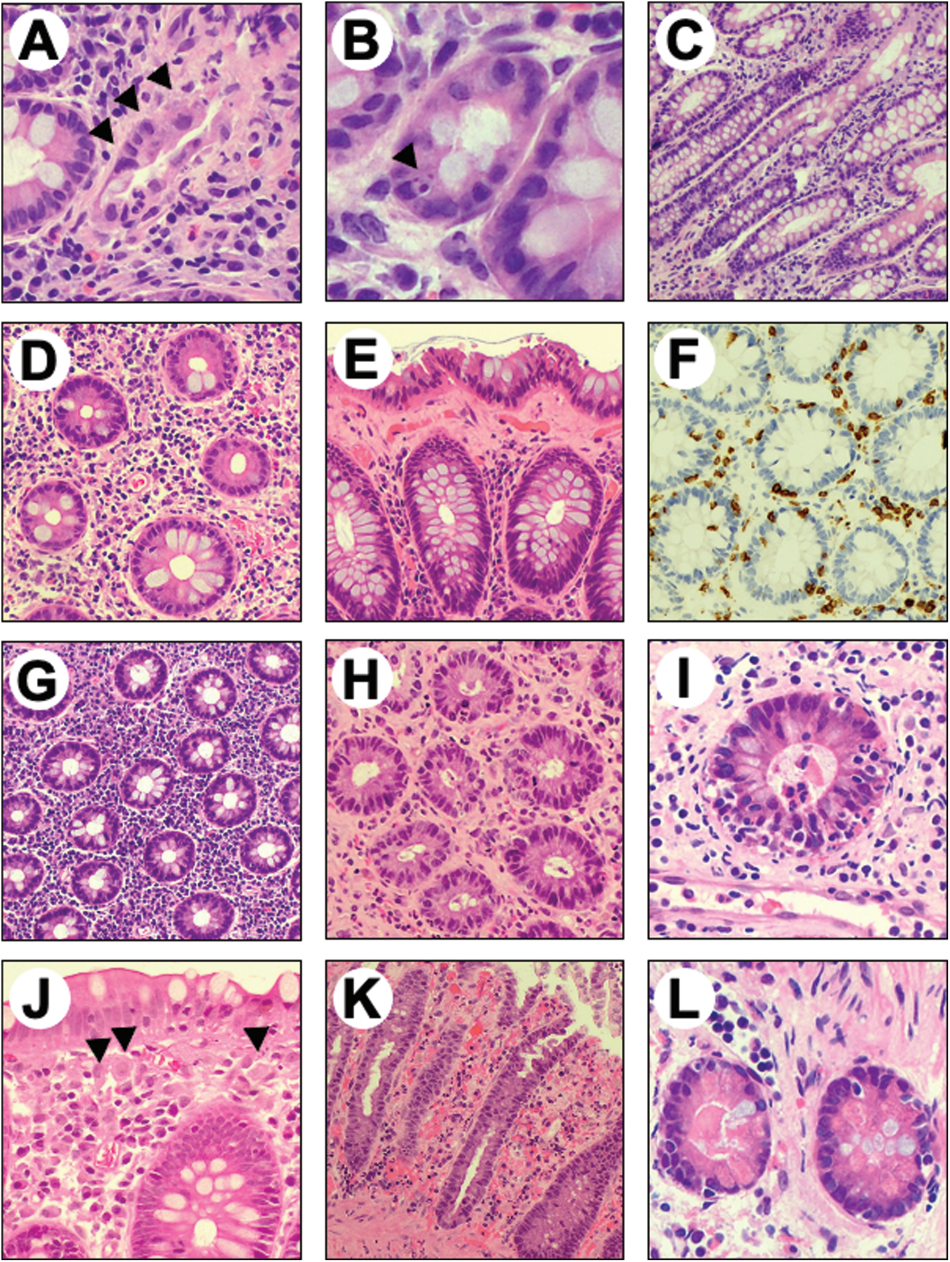
Representative examples of the microscopic alterations found in colic mucosa of patients treated with immune checkpoint inhibitors. (A) Crypt atrophy. (B) Apoptotic bodies within a gland. (C) Mild crypts’ distortion. (D) Lamina propria expansion and mucin depletion. (E) Collagenous band. (F). CD3 immunostaining showing moderate intraepithelial lympho-monocitic infiltrate. (G) Lympho-monocitic and plasmacellular infiltrate within the lamina propria. (H) Granulocytic infiltrate within the lamina propria associated with cryptitis. (I) Cryptic abscess. (J) Subepithelial macrophages. (K) Ischemic-like colitis features and superficial erosion. (L) Paneth metaplasia.

All colonic biopsies available at our institution were revised blinded and according to histological features, samples were classified as microscopic colitis in 7 out of 25 cases (28%) (Fig. 3). Collagenous colitis was present in 16% of revised cases (*n* = 4). Details about microscopic features of all revised colonic biopsies are reported in Table 3.

Among patients with macroscopic colitis, 3 out 11 (27.3%) were hospitalized versus 1 out of 19 (5.3%) without macroscopic alterations (*P* = .126).

Median duration of symptoms in patients with microscopic collagenous colitis was significantly longer than in the non-microscopic subgroup (median 200.5 days, IQR 118.5 -292.0; versus 54.0, IQR 30.0-101.5, *P* = .011).

### Budget Impact

Since the introduction of multidisciplinary evaluation of IMDC, an overall reduction in the costs spent to manage IMDC was observed. Specifically, the reduction was mainly related to the consistent decrease in hospitalizations (Table 2). In contrast, the rising in consumption of budesonide (+400%) and fecal calprotectin test (+143%) increased the related spending by more than 100%. To a lesser extent, the cost for colonoscopies also increased (+31%). However, compared with the previous period, we observed a reduction of ~20% in the annual cost (from 7.200 to 5.935 euros), with an even more pronounced reduction in the cost per patient (approximately −60%). The results are summarized in Supplementary Fig. S3.

## Discussion

Facing irAE is one of the main issues in the clinical management of advanced NSCLC. While international guidelines are mainly based on clinical trials experience and underline the importance of multidisciplinary approach, real-life experience analysis is essential to improve real-world patients’ quality of life and outcome.^20,22,25-28^

We retrospectively revised a large consecutive series of advanced patients with NSCLC treated with ICIs in monotherapy or in combination with chemotherapy and focused on clinical management and outcome of IMDC. In our real-world experience, incidence and outcome were similar to what expected according to literature data, especially for ICIs monotherapy^1,3-5,29-31^ and, as previously reported, the incidence of IMDC was associated with higher probability of prolonged PFS and OS among patients treated with immunotherapy.^24,32-34^ The correct interpretation of these results could be hindered by several biases such as a suboptimal diagnostic performance in the appropriate identification of irAE and immortal-time bias.^35,36^ To partially account for the latter, we have reported a landmark survival analysis at 12 weeks after the first ICI administration, which was consistent with the overall population.

As far as IMDC’s management is concerned, we noticed a relative low rate of colonoscopy examination and a long time between symptoms onset and endoscopic assessment, while its role has been recently highlighted and earlier colonoscopy (within 7 days) has been associated with better outcome.^17,37^ We also noticed a relatively longer duration of steroids administration, with respect to international guidelines’ indications, suggesting steroid tapering over 4-6 weeks and a limited use of second-line immunosuppressive drugs, which are still considered off-label in many European countries, including Italy.

On the contrary, the rate of IMDC relapse after treatment resumption seems to be slightly inferior to that reported in medical literature.^23^

The low rate of biologic therapies administered and the relatively long steroid duration in our cohort address the urgency to study-specific diagnostic-therapeutic pathways for IMDC and irAEs in general and potentially establishing specifically designed regulatory approval pathways as well.

Starting from real-world data observation, we focused on the impact of multidisciplinary management and analyzed our data according to the introduction of multidisciplinary discussion in our Cancer Institute, involving highly specialized gastroenterologists and pathologists. We selected diagnostic-therapeutic pathway indicators, according to literature data and consensus specifically concerning IMDC.^26,28^ After the introduction of multidisciplinary discussion, we noticed a statistically significant increase in colonoscopy and fecal calprotectin test. These aspects are supposed to impact significantly on outcome, based on literature data on IMDC. The role of colonoscopy in particular has been recently highlighted and its correlation with outcome significantly demonstrated.^38,39^ On the other side, fecal calprotectin test to personalize timing of ICIs resumption has been limited investigated in clinical practice but could be further evaluated on the basis of our experience, as fecal biomarker to monitor the course of IMDC.^40^ In parallel, after the introduction of MDT discussion, we started to use non-systemic steroids, which might limit steroids’ related toxicity and finally improve quality of life of patients, as previously reported.^41^ Further and prospective investigations are needed in order to identify the better imbrication method, dose and duration of systemic steroids after the introduction of budesonide.

In the frame of the project, we also included blinded revision of histological biopsies performed and noticed a relatively high rate of microscopic colitis, with respect to what expected in inflammatory bowel diseases but also in IMDC.^27,28,40,41^ We did not observe a statistically significant association between macroscopic endoscopic inflammation features and duration of symptoms, as recently described in a retrospective series including different solid tumors.^28^ However, it is worth mentioning that also in the quoted retrospective series there was no significant correlation between diarrhea grading and endoscopic features and that the literature series analyzing endoscopic and microscopic features of IMDC included patients treated with anti-CTLA4, likely to be associated with higher colitis severity.^17,27,28,38,42-44^

When we revised the microscopic features of our biopsies, we noticed a clinically significant correlation between collagenous features and duration of symptoms, thus suggesting that specific microscopic patterns could be associated with steroid-resistance independently on severity of symptoms at onset and underlining once more the role of systematic biopsy mapping during endoscopy. The possibility of early detection of grade 2 “steroid refractory” cases is likely to impact patients’ management, confirming the role of colonoscopy also in grade 2 IMDC, as indicated by international guidelines.^21,41,45-47^

We evaluated the change in outcome indicators after the introduction of multidisciplinary discussion and the most remarkable result concerns the reduction in the relapse rate after ICIs resumption. In these cases, we personalized management on the basis of clinical evaluation and fecal calprotectin test and in some cases, maintained budesonide, without systemic steroids at the time of ICIs resumption (20% of cases). Notably, we also noticed that after the introduction of multidisciplinary discussion, we have no case of symptoms grade increase after the detection of IMDC. Although not statistically significant, probably due to low number of patients hospitalized in the whole series, we observed 10 hospitalizations out of 58 cases before multidisciplinary discussion versus only one case out of 26 IMDC afterward.

Even though the number of patients included and the design of the study do not permit to draw definitive conclusion, we believe that outcome indicators improvement is related to the whole management improvement and, in particular, we believe that the use of calprotectin fecal test to monitor patients after ICI interruption and the use of non-systemic steroids are related to the reduction of relapse rate and even the reduction of grade worsening patients might be related to careful monitoring and customization of management according to diagnostic tools in addition to clinical presentation.

Further investigation, including prospective evaluation of diagnostic-therapeutic pathways and impact of multidisciplinary discussion is planned in order to evaluate long-term results and correlation between diagnostic-therapeutic pathways improvement and long-term results outcome, including evaluation of steroids’ side effects.

This study presents several strengths, as it depicts a large real-world series of advanced NSCLC patients treated according to clinical practice. By focusing on IMDC clinical management, we had the opportunity to collect broad clinical data regarding this relevant irAE and review histological samples from endoscopy and translational analyses are currently ongoing.

We acknowledge some limitations, including the retrospective and monocentric nature of this study and subsequent overall potential bias in reporting irAE-related symptoms, diagnostic procedures, and clinical management regarding complex clinical scenarios in a heterogenous series. Additionally, our patients were treated up to first ICIs' introduction in clinical practice and before the sedimentation of current diagnostic and clinical-management recommendations. Consistently, we have observed an overall suboptimal employment of diagnostic tools such as calprotectin and endoscopy, which was partially reverted after the introduction of an MDT discussion. Due to the number of patients included and the parallel impact of diagnostic pathway indicators changes, we cannot separately assess the direct correlation between each pathway indicator, including fecal calprotectin test and rate of colonoscopy, and outcome endpoints. In addition, we have not introduced yet institutional boards for multidisciplinary management of immune-related toxicities, as described in other experience.^11,48-50^

## Conclusion

Overall, the present study depicts a large real-world series of advanced patients with NSCLC treated according to clinical practice, focusing on IMDC clinical management, we had the opportunity to collect data about microscopic features in patients treated with anti-PD1/L1 and evaluate the impact of multidisciplinary discussion in clinical practice. The main limits are related to its retrospective nature, including the potential heterogeneity in reporting toxicity symptoms and planning diagnostic procedures. Nevertheless, the impact of multidisciplinary discussion, even in the absence of institutionalized tumor board, is remarkable and clearly indicates the need to change our clinical approach to IMDC and irAEs in general.

## Funding

The authors acknowledge Ricerca Corrente funding from the Italian Ministry of Health.

## Supplementary Material

oyad238_suppl_Supplementary_Material

## Data Availability

The data underlying this article will be shared on reasonable request to the corresponding author.

## References

[1] Brahmer J , Reckamp KL, Baas P, et al. Nivolumab versus docetaxel in advanced squamous-cell non–small-cell lung cancer. N Engl J Med. 2015;373(2):123-135. 10.1056/NEJMoa1504627 26028407 PMC4681400

[2] Borghaei H , Paz-Ares L, Horn L, et al. Nivolumab versus docetaxel in advanced nonsquamous non–small-cell lung cancer. N Engl J Med. 2015;373(17):1627-1639. 10.1056/NEJMoa1507643 26412456 PMC5705936

[3] Reck M , Rodríguez-Abreu D, Robinson AG, et al. Pembrolizumab versus chemotherapy for PD-L1–positive non–small-cell lung cancer. N Engl J Med. 2016;375(19):1823-1833. 10.1056/NEJMoa1606774 27718847

[4] Herbst RS , Baas P, Kim D-W, et al. Pembrolizumab versus docetaxel for previously treated, PD-L1-positive, advanced non-small-cell lung cancer (KEYNOTE-010): a randomised controlled trial. Lancet 2016;387(10027):1540-1550. 10.1016/S0140-6736(15)01281-7 26712084

[5] Rittmeyer A , Barlesi F, Waterkamp D, et al.. Atezolizumab versus docetaxel in patients with previously treated non-small-cell lung cancer (OAK): a phase 3, open-label, multicentre randomised controlled trial. Lancet 2017;389(10066):255-265. 10.1016/S0140-6736(16)32517-X 27979383 PMC6886121

[6] Friedman CF , Proverbs-Singh TA, Postow MA. Treatment of the immune-related adverse effects of immune checkpoint inhibitors: a review. JAMA Oncol. 2016;2(10):1346-1353. 10.1001/jamaoncol.2016.1051 27367787

[7] Faivre-Finn C , Vicente D, Kurata T, et al. Four-year survival with durvalumab after chemoradiotherapy in stage III NSCLC—an update from the PACIFIC trial. J Thorac Oncol. 2021;16(5):860-867. 10.1016/j.jtho.2020.12.015 33476803

[8] Wakelee HA , Altorki NK, Zhou C, et al. IMpower010: Primary results of a phase III global study of atezolizumab versus best supportive care after adjuvant chemotherapy in resected stage IB-IIIA non-small cell lung cancer (NSCLC). J Clin Oncol. 2021;39(15_suppl):8500-8500. 10.1200/jco.2021.39.15_suppl.8500

[9] Wang DY , Salem J-E, Cohen JV, et al. Fatal toxic effects associated with immune checkpoint inhibitors: a systematic review and meta-analysis. JAMA Oncol. 2018;4(12):1721-1728. 10.1001/jamaoncol.2018.3923 30242316 PMC6440712

[10] Ahern E , Allen MJ, Schmidt A, Lwin Z, Hughes BGM. Retrospective analysis of hospital admissions due to immune checkpoint inhibitor-induced immune-related adverse events (irAE). Asia Pac J Clin Oncol. 2021;17(2):e109-e116. 10.1111/ajco.13350 32519444

[11] Balaji A , Zhang J, Marrone K, et al. Immune-related adverse events requiring inpatient management: spectrum of toxicity, treatment, and outcomes. J Clin Oncol. 2018;36(5_suppl):138-138. 10.1200/jco.2018.36.5_suppl.138

[12] Reynolds KL , Cohen JV, Durbin S, et al. Inpatient admissions related to immune-related adverse effects (irAE) among patients treated with immune checkpoint inhibitors for advanced malignancy: a tsunami is coming, but are we ready?. J Clin Oncol. 2018;36(5_suppl):127-127. 10.1200/jco.2018.36.5_suppl.127

[13] Chu JN , Choi JG, Ostvar S, et al. Cost of inpatient admissions for immune-related adverse effects from immune checkpoint inhibitor therapy: a single center experience. J Clin Oncol. 2018;36(15_suppl):3060-3060. 10.1200/jco.2018.36.15_suppl.306030188785

[14] Pasello G , Pavan A, Attili I, et al. Real world data in the era of immune checkpoint inhibitors (ICIs): increasing evidence and future applications in lung cancer. Cancer Treat Rev. 2020;87:102031. 10.1016/j.ctrv.2020.102031 32446182

[15] Lee SM , Schulz C, Prabhash K, et al. LBA11 IPSOS: results from a phase III study of first-line (1L) atezolizumab (atezo) vs single-agent chemotherapy (chemo) in patients (pts) with NSCLC not eligible for a platinum-containing regimen. Ann Oncol. 2022;33(Suppl 7):S1418-S1419. 10.1016/j.annonc.2022.08.052

[16] Paz-Ares L , Luft A, Vicente D, et al.. Pembrolizumab plus chemotherapy for squamous non–small-cell lung cancer. N Engl J Med. 2018;379(21):2040-2051. 10.1056/NEJMoa1810865 30280635

[17] Gong Z , Wang Y. Immune checkpoint inhibitor–mediated diarrhea and colitis: a clinical review. JCO Oncol Pract. 2020;16(8):453-461. 10.1200/OP.20.00002 32584703

[18] Weber JS , Kähler KC, Hauschild A. Management of immune-related adverse events and kinetics of response with ipilimumab. J Clin Oncol. 2012;30(21):2691-2697. 10.1200/JCO.2012.41.6750 22614989

[19] Wang DY , Mooradian MJ, Kim DW, et al. Clinical characterization of colitis arising from anti-PD-1 based therapy. Oncoimmunology 2019;8(1):1-8. 10.1080/2162402X.2018.1524695 PMC6287774 30546965

[20] Haanen JBAG , Carbonnel F, Robert C, et al.. Management of toxicities from immunotherapy: ESMO Clinical Practice Guidelines for diagnosis, treatment and follow-up. Ann Oncol. 2017;28(suppl_4):iv119-iv142. 10.1093/annonc/mdx225 28881921

[21] Brahmer JR , Lacchetti C, Schneider BJ, et al.. Management of immune-related adverse events in patients treated with immune checkpoint inhibitor therapy: American Society of Clinical Oncology Clinical Practice Guideline. J Clin Oncol. 2018;36(17):1714-1768. 10.1200/JCO.2017.77.6385 29442540 PMC6481621

[22] Brahmer JR , Abu-Sbeih H, Ascierto PA, et al. Society for immunotherapy of cancer (sitc) clinical practice guideline on immune checkpoint inhibitor-related adverse events. J ImmunoTher Cancer. 2021;9(6):e002435. 10.1136/jitc-2021-002435 34172516 PMC8237720

[23] Abu-Sbeih H , Ali FS, Naqash AR, et al. Resumption of immune checkpoint inhibitor therapy after immune-mediated colitis. J Clin Oncol. 2019;37(30):2738-2745. 10.1200/JCO.19.00320 31163011 PMC6800279

[24] Pavan A , Calvetti L, Dal Maso A, et al. Peripheral blood markers identify risk of immune-related toxicity in advanced non-small cell lung cancer treated with immune-checkpoint inhibitors. Oncologist 2019;24(8):1128-1136. 10.1634/theoncologist.2018-0563 31015312 PMC6693718

[25] Schneider BJ , Naidoo J, Santomasso BD, et al. Management of immune-related adverse events in patients treated with immune checkpoint inhibitor therapy: ASCO guideline update. J Clin Oncol. 2021;39(36):4073-4126. 10.1200/JCO.21.01440 34724392

[26] Nahar KJ , Rawson RV, Ahmed T, et al. Clinicopathological characteristics and management of colitis with anti-PD1 immunotherapy alone or in combination with ipilimumab. J ImmunoTher Cancer. 2020;8(2):e001488-e001412. 10.1136/jitc-2020-001488 33234603 PMC7689081

[27] Chen JH , Pezhouh MK, Lauwers GY, Masia R. Histopathologic features of colitis due to immunotherapy with anti-PD-1 antibodies. Am J Surg Pathol. 2017;41(5):643-654. 10.1097/PAS.0000000000000829 28296676

[28] Geukes Foppen MH , Rozeman EA, van Wilpe S, et al. Immune checkpoint inhibition-related colitis: symptoms, endoscopic features, histology and response to management. ESMO Open 2018;3(1):e000278-e000278. 10.1136/esmoopen-2017-000278 29387476 PMC5786923

[29] Figueiredo A , Almeida MA, Almodovar MT, et al. Real-world data from the Portuguese Nivolumab Expanded Access Program (EAP) in previously treated Non Small Cell Lung Cancer (NSCLC). Pulmonology 2020;26(1):10-17. 10.1016/j.pulmoe.2019.06.001 31630986

[30] Areses Manrique M. C , Mosquera Martínez J, García González J, et al. Real world data of nivolumab for previously treated non-small cell lung cancer patients: a Galician lung cancer group clinical experience. Transl Lung Cancer Res. 2018;7(3):404-415. 10.21037/tlcr.2018.04.03 30050778 PMC6037977

[31] Brustugun OT , Sprauten M, Helland. Real-world data on nivolumab treatment of non-small cell lung cancer. Acta Oncol. (Madr). 2017;56(3):438-440. 10.1080/0284186X.2016.1253865 27892773

[32] Haratani K , Hayashi H, Chiba Y, et al. Association of immune-related adverse events with nivolumab efficacy in non-small cell lung cancer. JAMA Oncol. 2018;4(3):374-378. 10.1001/jamaoncol.2017.2925 28975219 PMC6583041

[33] Hsiehchen D , Naqash AR, Espinoza M, et al. Association between immune-related adverse event timing and treatment outcomes. Oncoimmunology 2022;11(1):1-8. 10.1080/2162402X.2021.2017162 PMC8741287 35003896

[34] Weingarden AR , Gubatan J, Singh S, et al. Immune checkpoint inhibitor-mediated colitis is associated with cancer overall survival. World J Gastroenterol. 2022;28(39):5750-5763. 10.3748/wjg.v28.i39.5750 36338892 PMC9627421

[35] Dall’Olio FG , Di Nunno V, Massari F. Immortal time bias question in the association between toxicity and outcome of immune checkpoint inhibitors. J Clin Oncol. 2020;38(1):105-106. 10.1200/JCO.19.01728 31675246

[36] Hsiehchen D , Watters MK, Lu R, Xie Y, Gerber DE. Variation in the assessment of immune-related adverse event occurrence, grade, and timing in patients receiving immune checkpoint inhibitors. JAMA Netw Open 2019;2(9):e1911519. 10.1001/jamanetworkopen.2019.11519 31532516 PMC6751757

[37] Abu-Sbeih H , Ali FS, Alsaadi D, et al. Outcomes of vedolizumab therapy in patients with immune checkpoint inhibitor-induced colitis: a multi-center study 11 Medical and Health Sciences 1103 Clinical Sciences. J ImmunoTher Cancer. 2018;6(1):1-11. 30518410 10.1186/s40425-018-0461-4 PMC6280383

[38] Abu-Sbeih H , Ali FS, Luo W, et al. Importance of endoscopic and histological evaluation in the management of immune checkpoint inhibitor-induced colitis 11 Medical and Health Sciences 1103 Clinical Sciences. J ImmunoTher Cancer. 2018;6(1):1-11. 30253811 10.1186/s40425-018-0411-1 PMC6156850

[39] Yamauchi Y , Arai M, Akizue N, et al. Colonoscopic evaluation of diarrhea/colitis occurring as an immune-related adverse event. Jpn J Clin Oncol. 2021;51(3):363-370. 10.1093/jjco/hyaa203 33290513

[40] Zou F , Wang X, Glitza Oliva IC, et al. Fecal calprotectin concentration to assess endoscopic and histologic remission in patients with cancer with immune-mediated diarrhea and colitis. J ImmunoTher Cancer. 2021;9(1):e002058-e002058. 10.1136/jitc-2020-002058 33436487 PMC7805368

[41] Hughes MS , Molina GE, Chen ST, et al. Budesonide treatment for microscopic colitis from immune checkpoint inhibitors. J ImmunoTher Cancer. 2019;7(1):1-10. 31699151 10.1186/s40425-019-0756-0 PMC6839080

[42] Khoja L , Day D, Wei-Wu Chen T, Siu LL, Hansen AR. Tumour- and class-specific patterns of immune-related adverse events of immune checkpoint inhibitors: a systematic review. Ann Oncol. 2017;28(10):2377-2385. 10.1093/annonc/mdx286 28945858

[43] Som A , Mandaliya R, Alsaadi D, et al. Immune checkpoint inhibitor-induced colitis: a comprehensive review. World J Clin. Cases 2019;7(4):405-418. 10.12998/wjcc.v7.i4.405 30842952 PMC6397821

[44] Parente P , Maiorano BA, Ciardiello D, et al. Clinic, endoscopic and histological features in patients treated with ICI developing GI toxicity: some news and reappraisal from a mono-institutional experience. Diagnostics 2022;12(3):685-612. 10.3390/diagnostics12030685 35328239 PMC8947154

[45] Fredrick TW , Ramos GP, Braga Neto MB, et al. Clinical course and impact of immune checkpoint inhibitor colitis resembling microscopic colitis. Crohns Colitis 360 2022;4(2):1-4. 10.1093/crocol/otac008 PMC9802423 36777041

[46] Thompson JA , Schneider BJ, Brahmer J, et al. NCCN guidelines insights: management of immunotherapy-related toxicities, version 1.2020. J Natl Compr Canc Netw. 2020;18(3):231-241. 10.6004/jnccn.2020.0012 32135517

[47] Haanen J , Obeid M, Spain L, et al. Management of toxicities from immunotherapy: ESMO Clinical Practice Guideline for diagnosis, treatment and follow-up. Ann Oncol. 2022;33(12):1217-1238. 10.1016/j.annonc.2022.10.001 36270461

[48] Kennedy LC , Wong KM, Kamat NV, et al. Untangling the multidisciplinary care web: streamlining care through an immune-related adverse events (IRAE) tumor board. Target Oncol. 2020;15(4):541-548. 10.1007/s11523-020-00739-5 32710246 PMC7489785

[49] Läubli H , Dirnhofer S, Zippelius A. Immune tumor board: integral part in the multidisciplinary management of cancer patients treated with cancer immunotherapy. Virchows Arch. 2019;474(4):485-495. 10.1007/s00428-018-2435-9 30143868

[50] Michot JM , Lappara A, Le Pavec J, et al. The 2016-2019 ImmunoTOX assessment board report of collaborative management of immune-related adverse events, an observational clinical study. Eur J Cancer. 2020;130:39-50. 10.1016/j.ejca.2020.02.010 32172197

