# Supplementary material for: Immune-Related Diarrhea and Colitis in Non-small Cell Lung Cancers: Impact of Multidisciplinary Management in a Real-World Setting
Source: Oncologist. 2023 Aug 21;29(1):e118–30. doi: 10.1093/oncolo/oyad238 (PMC10769780; doi:10.1093/oncolo/oyad238)
Supplement: oyad238_suppl_Supplementary_Material [file oyad238_suppl_supplementary_material.zip › Supplementary Figure Captions.docx]

**Supplementary Figure Captions**

**Supplementary Figure 1**

Landmark analysis at 12 weeks for progression free survival (PFS, A) and overall survival (OS, B) in patients treated with ICIs according to the presence of immune-mediated diarrhea and colitis (ICDM).

**Supplementary Figure 2**

Clinical impact of definitive interruption of ICIs among patients experiencing IMDC, according to definitive interrupt (in terms of progression-free survival, A and overall survival, B)

**Supplementary Figure 3**

Change (%) in costs after the introduction of multidisciplinary evaluation of IMDC
