# Supplementary material for: Immune-Related Diarrhea and Colitis in Non-small Cell Lung Cancers: Impact of Multidisciplinary Management in a Real-World Setting
Source: Oncologist. 2023 Aug 21;29(1):e118–30. doi: 10.1093/oncolo/oyad238 (PMC10769780; doi:10.1093/oncolo/oyad238)
Supplement: oyad238_suppl_Supplementary_Material [file oyad238_suppl_supplementary_material.zip › Supplementary Tables.docx]

**Supplementary Table 1. Patients’ characteristics and treatments**

| **Variable** | | | ***N* (%)** | |
| --- | --- | --- | --- | --- |
|  |  |  |  |  |
| **Number of cases** | | | 607 | (100.0) |
| **Age (years), median (range)** | | | 68.7 | 37-89 |
| **Gender** | | |  |  |
|  | Male | | 398 | (65.5) |
|  | Female | | 209 | (34.5) |
| **Smoking status** | | |  |  |
|  | Never smokers | | 92 | (15.1) |
|  | Former smokers | | 317 | (52.2) |
|  | Smokers | | 198 | (32.6) |
| **Tumor histology** | | |  |  |
|  | Adenocarcinoma | | 394 | (64.9) |
|  | Squamous | | 144 | (23.7) |
|  | Other | | 69 | (11.4) |
| **PD-L1 (Tumor Proportion Score)** | | |  |  |
|  | ≥50% | | 219 | (36.1) |
|  | 1-49% | | 120 | (12.0) |
|  | <1% | | 178 | (29.3) |
|  | Not available | | 90 | (14.9) |
| **ECOG PS at treatment start** | | |  |  |
|  | 0 | | 113 | (18.6) |
|  | 1 | | 396 | (65.2) |
|  | ≥2 | | 98 | (16.1) |
| **Number of metastatic sites at diagnosis** | | | |  |
|  | <3 | | 467 | (76.9) |
|  | ≥3 | | 140 | (23.1) |
| **Stage** | | |  |  |
|  | IIIB-C | | 8 | (1.3%) |
|  | IV | | 599 | (98.7) |
| **Treatment received** | | |  |  |
|  | ICIs single agent | | 483 | (79.6%) |
|  | | Nivolumab | 162 | (26.7) |
|  | | Pembrolizumab | 233 | (38.4) |
|  | | Atezolizumab | 88 | (14.5) |
|  | Pembrolizumab plus chemotherapy (1^st^ line) | | 124 | (20.4) |

Abbreviations: N, number of cases; PD-L1, programmed death-ligand 1; PS, performance status; ECOG, Eastern Cooperative Oncology Group; ICIs, immune-checkpoint inhibitors.

**Supplementary Table 2. Patients’ outcome in the all populations and according to treatment received**

| **Variable** | **All population** | **Pembrolizumab plus chemotherapy First Line** | **ICIs**  **First line** | **ICIs**  **Subsequent line** | ***p**** | ***p***** | ***p****** |
| --- | --- | --- | --- | --- | --- | --- | --- |
|  | ***N* (%)** | ***N* (%)** | ***N* (%)** | ***N* (%)** |  |  |  |
| **Number of cases** | 607 (100) | 124 (100) | 184 (100) | 299 (100) | - | - | -. |
| **Response Rate** | 182 (30.0) | 63 (50.8) | 79 (42.9) | 40 (13.4) | 0.214 | <0.001 | <0.001 |
| **Disease control rate** | 361 (59.5) | 96 (77.4) | 124 (67.4) | 141 (47.1) | 0.075 | <0.001 | <0.001 |
| **PFS months (median, 95% CI)** | 7.7 (6.6-8.9) | 10.1 (7.2-13.0) | 10.8 (6.2- 15.3) | 4.3 (3.3-5.3) | 0.247 | <0.001 | <0.001 |
| **OS months**  **(median, 95% CI)** | 10.9 (9.4-12.4) | 16.8 (12.2-21.4) | 15.4 (11.5-19.3) | 7.5 (5.9-9.1) | 0.389 | <0.001 | <0.001 |

Abbreviations: N, number; ICIs, immune-checkpoint inhibitors; PFS, progression free survival; OS, overall survival, calculated from the start of the study treatment.

* Comparison between pembrolizumab *plus* chemotherapy first line *versus* ICIs first-line.

**Comparison between pembrolizumab *plus* chemotherapy first line *versus* ICIs subsequent line.

***Comparison between pembrolizumab first line *versus* ICIs subsequent line.

Statistical analysis: log-rank test for survivals; chi-square for ratios.

**Supplementary Table 3.** **Univariate (log-rank) and multivariate (cox-proportional hazard) analysis for progression-free survival and overall survival in all patients.**

| **Variable** | | ***N* (%)** | | **PFS**  **Univariate analysis** | **PFS**  **Multivariate analysis** | | | **OS**  **Univariate analysis** | **OS**  **Multivariate analysis** | | |
| --- | --- | --- | --- | --- | --- | --- | --- | --- | --- | --- | --- |
|  |  |  |  | ***p*** | **Coefficient** | ***p*** | **HR (95% CI)** | ***p*** | **Coefficient** | ***p*** | **HR (95%CI)** |
| **Number of cases** | | 607 | (100) |  |  |  |  |  |  |  |  |
| **Gender** | |  |  |  |  |  |  |  |  |  |  |
|  | Male | 398 | (65.5) | 0.513 | -0.0599 | 0.584 | 0.942 (0.760 - 1.167) | 0.176 | -0.0899 | 0.415 | 0.914 (0.737 - .134) |
|  | Female | 209 | (34.5) |  |  |  |  |  |  |  |  |
| **Smoking status** | |  |  |  |  |  |  |  |  |  |  |
|  | Smokers | 515 | (84.9) | 0.159 | 0.225 | 0.100 | 1.253 (0.958-1.639) | 0.763 | 0.135 | 0.335 | 1.145 (0.870 - 1.506) |
|  | Never smokers | 92 | (15.1) |  |  |  |  |  |  |  |  |
| **PD-L1 status** | |  |  |  |  |  |  |  |  |  |  |
|  | < 1% | 180 | (29.7) | **<0.001** | **0.488** | **<0.001** | **1.628 (1.298 - 2.042)** | **<0.001** | **0.463** | **<0.001** | **1.589 (1.271 - 1.985)** |
|  | ≥ 1% | 339 | (55.8) |  |  |  |  |  |  |  |  |
|  | Unknown | 88 | (14.5) |  |  |  |  |  |  |  |  |
| **Histology** | | | |  |  |  |  |  |  |  |  |
|  | Adeno | 394 | (64.9) | 0.072 | - | **-** | - | **<0.001** | **-0.446** | **<0.001** | **0.640 (0.504 0.813)** |
|  | Squamous | 144 | (23.7) |  |  |  |  |  |  |  |  |
|  | Other | 69 | (11.4) |  |  |  |  |  |  |  |  |
| **Age** | |  |  |  |  |  |  |  |  |  |  |
|  | < 68 | 283 | (39.2) | 0.800 | 0.0175 | 0.866 | 1.018 (0.831-1.247) | **0.019** | 0.182 | 0.077 | 1.199 (0.980 - 1.467) |
|  | ≥ 68 | 324 | (53.2) |  |  |  |  |  |  |  |  |
| **N met site at diagnosis** | | |  |  |  |  |  |  |  |  |  |
|  | 0-1 | 264 | (43.5) | **<0.001** | **0.543** | **<0.001** | **1.721 (1.393 - 2.125)** | **<0.001** | **0.486** | **<0.001** | **1.626 (1.326 - 1.994)** |
|  | >1 | 343 | (56.5) |  |  |  |  |  |  |  |  |
| **PS** | |  |  |  |  |  |  |  |  |  |  |
|  | 0-1 | 509 | (83.9) | **<0.001** | **0.904** | **<0.001** | **2.469 (1.890-3.227)** | **<0.001**- | **1.209** | **<0.001** | **3.351 (2.601 - 4.319)** |
|  | >1 | 98 | (16.1) |  |  |  |  |  |  |  |  |
| **IMDC** | |  |  |  |  |  |  |  |  |  |  |
|  | Present | 84 | (13.8) | **<0.001** | **-0.705** | **<0.001** | **0.494 (0.358 - 0.681)** | **<0.001**- | **-0.695** | **<0.001** | **0.499 (0.359 - 0.695)** |
|  | Absent | 523 | (86.2) |  |  |  |  |  |  |  |  |
| **Lombosacral RT** | |  |  |  |  |  |  |  |  |  |  |
|  | Present | 68 | (11.2) | **0.016** | 0.255 | 0.088 | 1.291 (0.963-1.730) | 0.148 | 0.118 | 0.427 | 1.125 (0.842 - 1.503) |
|  | Absent | 539 | (88.8) |  |  |  |  |  |  |  |  |

Abbreviations: N, number, PFS, progression-free survival; OS, overall survival; HR, hazard ratio; CI, confidence interval; met, metastatic; PS, performance status; IMDC, immune-mediated diarrhea and colitis; RT, radiotherapy.

S**upplementary table 4.** **Clinical features in patients with immune-related diarrhea and colitis in whole study population and according to the treatment received**

| **Variable** | | | **All population**  ***N* (%)** | | **ICI plus ChT First Line**  ***N* (%)** | | **ICIs  Any line**  ***N* (%)** | | ***p§*** |
| --- | --- | --- | --- | --- | --- | --- | --- | --- | --- |
| **Diarrhea cases** | | | 84 | (100) | 19 | (100) | 65 | (100) |  |
| **Age (years), median (range)** | | | 69 | (38.3-88.7) | 67.9 | (38.3-88.7) | 69 | (50-77) | 0.987 |
| **PS at treatment start** | | |  |  |  |  |  |  |  |
|  | | **0-1** | 77 | (91.6) | 19 | (100) | 58 | (89.2) | -- |
|  | | **>1** | 7 | (8.3) | 0 | (0.0) | 7 | (10.8) |  |
| **Grade at onset** | | |  |  |  |  |  |  |  |
|  | G1-G2 | | 77 | (91.7) | 19 | (100) | 58 | (89.2) | 0.568 |
|  | G3 | | 7 | (83.3 | 0 | (0.0) | 7 | (10.8) |  |
| **Max grade** | | |  |  |  |  |  |  |  |
|  | G1-G2 | | 70 | (83.3) | 17 | (89.5) | 53 | (81.5) | 0.508 |
|  | G3 | | 14 | (16.7) | 2 | (10.5) | 12 | (18.4) |  |
| **Time to symptoms onset** | | |  |  |  |  |  |  |  |
|  | Median, IQ range | | 3.34 | (1.530-7.32) | 3.684 | (1.63-5.07) | 3.191 | (1.4-7.7) | 0.597 |
| **Associated symptoms** | | |  |  |  |  |  |  |  |
|  | Present | | 34 | (40.5) | 9 | (47.3) | 25 | (38.5) | 0.667 |
|  | Absent | | 50 | (59.5) | 10 | (52.7) | 40 | (61.5) |  |
| **Treatment interruption** | | |  |  |  |  |  |  |  |
|  | Yes | | 59 | (70.2) | 14 | (73.7) | 45 | (69.2) | 0.281 |
|  | No | | 25 | (29.8) | 5 | (26.3) | 20 | (30.8) |  |
| **Steroid treatment** | | |  |  |  |  |  |  |  |
|  | Yes | | 68 | (81.0) | 15 | (78.9) | 53 | (81.5) |  |
|  | No | | 16 | (19.0) | 4 | (21.0) | 5 | (18.5) |  |
| **Steroid duration** | | |  |  |  |  |  |  |  |
|  | Median, IQ range | | 122.5 | (75.0-252.0) | 131.0 | (72.0-156.5) | 121.0. | (75-294) | 0.921 |
| **Max steroid dose** | | |  |  |  |  |  |  |  |
|  | <1 mg/kg * | | 53 | (63.1) | 12 | (63.2) | 41 | (63.1) | 1.000 |
| **.** | >1 mg/kg * | | 16 | (19.0) | 4 | (21.1) | 12 | (18.5) |  |
|  | Not Applicable | | 15 | (17.8) | 3 | (15.8) | 12 | (18.5) |  |
| **Time to symptoms resolution** | | |  |  |  |  |  |  |  |
|  | Median, IQ range | | 48.0 | (24.-91.5) | 55.5 | (34.0-99.5) | 45.0. | (25.3-64.8) | 0.174 |
| **Time to coloscopy** | | |  |  |  |  |  |  |  |
|  | Median, IQ range | | 55.0 | (28.5-128.5) | 83.5 | (33.0-131.0) | 50.0 | (19.5-75.7) | 0.203 |
| **Treatment resumption** | | |  |  |  |  |  |  |  |
|  | Yes | | 30 | (35.7) | 6 | (31.6) | 24 | (37.0) | 0.705 |
|  | No | | 29 | (34.5) | 8 | (42.1) | 21 | (32.3) |  |
|  | Not applicable | | 25 | (29.7) | 5 | (26.3) | 20 | (30.8) |  |
| **Time to treatment resumption** | | |  |  |  |  |  |  |  |
|  | Median, 95% CI (mo) | | 4.9 | (-0.4-10.2) | 14.9 | (- 0.8-30.7) | 4.7 | (0-9.5) | 0.291 |
| **Diarrhea recurrence after ICIs reintroduction** | | |  |  |  |  |  |  |  |
|  | Yes | | 19 | (22.6) | 4 | (21.0) | 15 | (23.0) | 1.000 |
|  | No | | 11 | (13.1) | 2 | (10.5) | 9 | (13.8) |  |
|  | Not applicable | | 54 | (64.3) | 13 | (68.4) | 41 | (63.1) |  |
| **Time to recurrence** | | |  |  |  |  |  |  |  |
|  | Median, 95%CI (mo) | | 2.1 | (1.1-3.0) | - |  | - |  | -- |
| **Hospitalization** | | |  |  |  |  |  |  |  |
|  | Yes | | 11 | (13.1) | 1 | (5.3) | 10 | (15.4) | 0.442 |
|  | No | | 73 | (86.9) | 18 | (94.7) | 55 | (84.6) |  |
| **Hospitalization Time** | | |  |  |  |  |  |  |  |
|  | Median, IQ range | | 15.5 | (9.0-17.0) | 3.0 | (3.0-3.0) | 16.0 | (9.8-17.3) | 0.162 |

Abbreviations: N, number; ICIs, immune-checkpoint inhibitors; G, grade; CI, confidence interval; IQ, interquartile; mo, months. *dose in mg/kg methylprednisolone equivale.

§ Kaplan-meyer method and log-rank test for variables with censored cases; Mann-Witney rank sum test for continuous variables without censored values.

**Supplementary table 5. Clinical features of patients who resume treatments according to relapsing status.**

| **Variable** | | **IMDC relapsing**  ***N* (%)** | | **No IMDC relapsing**  ***N* (%)** | | ***p*** |
| --- | --- | --- | --- | --- | --- | --- |
| **All cases** | | 19 | (100) | 11 | (100) |  |
| **Budesonide** | |  |  |  |  |  |
|  | Yes | 4 | (21.1) | 5 | (45.5) | 0.225 |
|  | No | 15 | (78.9) | 6 | (54.5) |  |
| **Fecal calprotectin test** | |  |  |  |  |  |
|  | Yes | 4 | (21.1) | 4 | (36.4) | 0.417 |
|  | No | 15 | (78.9) | 7 | (63.6) |  |
| **Colonoscopy** | |  |  |  |  |  |
|  | Yes | 4 | (21.1) | 6 | (54.5) | 0.108 |
|  | No | 15 | (78.9) | 5 | (45.5) |  |
| **Gastroenterological visit** | |  |  |  |  |  |
|  | Yes | 3 | (15.8) | 4 | (36.4) | 0.372 |
|  | No | 16 | (82.2) | 7 | (63.6) |  |
| **Max grade** | |  |  |  |  |  |
|  | G1-2 | 14 | (73.7) | 9 | (81.8) | 1.0 |
|  | G3 | 5 | (26.3) | 2 | (18.2) |  |
| **Hospitalization** | |  |  |  |  |  |
|  | Yes | 2 | (10.5) | 0 | (0.0) | 0.520 |
|  | No | 17 | (89.5) | 11 | (100.0) |  |
