# Supplementary figures and images for: Immune-Related Diarrhea and Colitis in Non-small Cell Lung Cancers: Impact of Multidisciplinary Management in a Real-World Setting
Source: Oncologist. 2023 Aug 21;29(1):e118–30. doi: 10.1093/oncolo/oyad238 (PMC10769780; doi:10.1093/oncolo/oyad238)

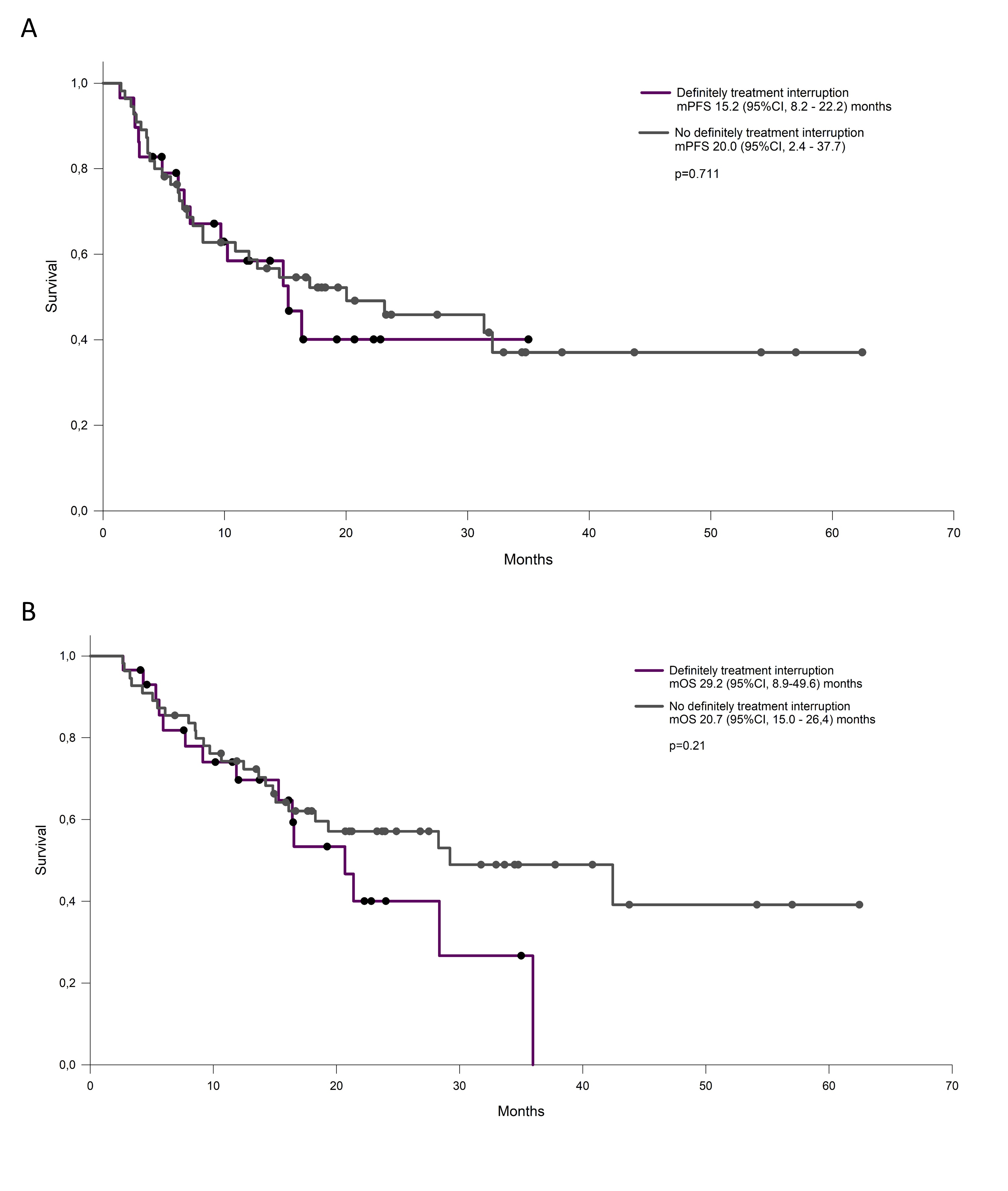

Supplement: oyad238_suppl_Supplementary_Material [file oyad238_suppl_supplementary_material.zip › Supplementary Figure 1.tif]

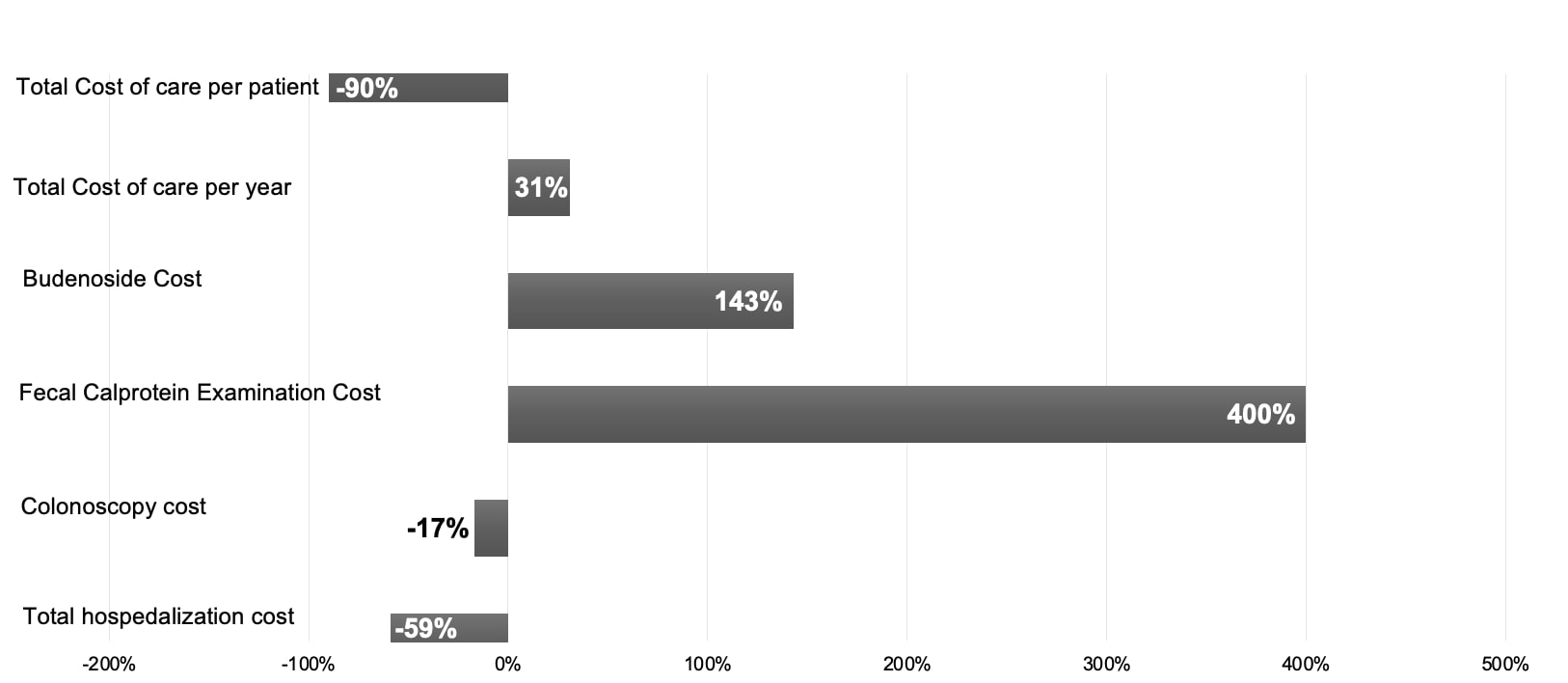

Supplement: oyad238_suppl_Supplementary_Material [file oyad238_suppl_supplementary_material.zip › Supplementary Figure 2.jpg]

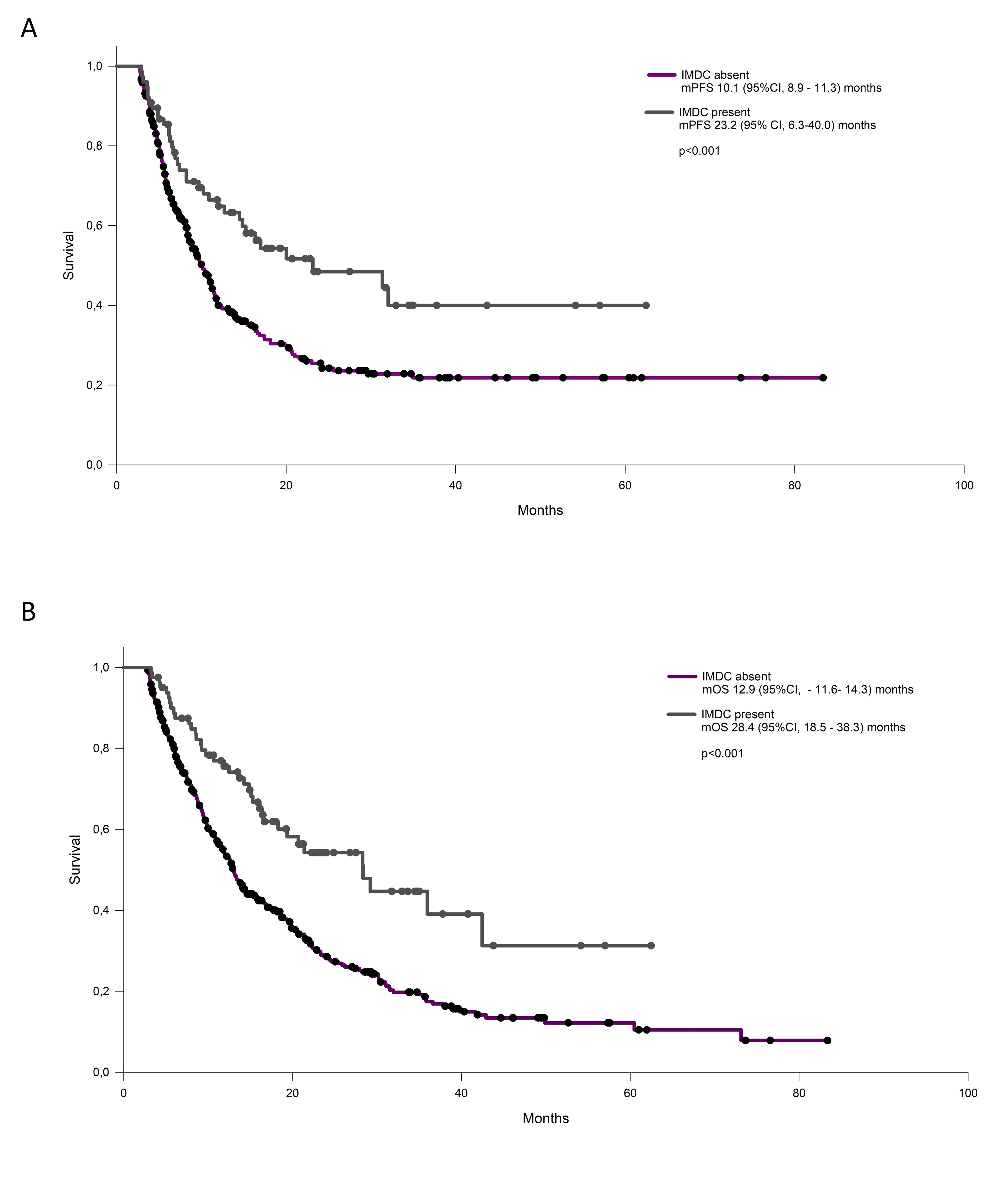

Supplement: oyad238_suppl_Supplementary_Material [file oyad238_suppl_supplementary_material.zip › Supplementary figure 3.tif]
